# Incremental Healthcare Costs and Outpatient Antifungal Treatment of Patients with Aspergillosis in the United States

**DOI:** 10.36469/9862

**Published:** 2013-10-08

**Authors:** Emily Durden, Donna McMorrow, Paul Juneau, Robert Fowler, Paresh Chaudhari, David Horn

**Affiliations:** 1 Truven Health Analytics, Austin TX, USA; 2 Astellas Scientific and Medical Affairs, Inc., Northbrook, IL, USA

**Keywords:** treatment, costs, burden, economic, aspergillosis

## Abstract

**Objectives:** To evaluate the total and outpatient economic burden of aspergillosis, and to describe the outpatient antifungal treatment of aspergillosis within a large, commercially-insured population in the United States.

**Methods:** Adults with at least one medical claim with an aspergillosis diagnosis (International Classification of Disease 9th Revision Clinical Modification [ICD-9-CM] code 117.3 or 484.6) between 07/01/04-03/01/11 were identified from the MarketScan Research Databases. Patients had ≥6 months of pre-index and ≥1 month of post-index continuous health plan and pharmacy benefit enrollment and no pre-index diagnosis of aspergillosis. Aspergillosis cases were propensity score-matched to a sample of controls without aspergillosis. Outpatient antifungal therapy and total and outpatient healthcare resource utilization were evaluated in the post-index period. General linear models were used to estimate costs, which were adjusted by the length of follow-up. Incremental costs were calculated between cohorts and a bootstrap procedure was used to produce corresponding variation and 95% confidence interval estimates.

**Results:** Aspergillosis cases (N=5,499; mean age: 57.8 years; 48.6% female; 64.2% with cancer) were matched to 5,499 controls (mean age: 58.3 years; 48.4% female; 60.6% with cancer). Two-thirds of the aspergillosis cases had no outpatient prescription for an antifungal within 30 days of index; for those with outpatient antifungal therapy, voriconazole was the most commonly prescribed agent (60.9%). Average adjusted total and outpatient expenditures were greater for aspergillosis patients during follow-up than those of the matched controls ($26,680 and $9,248 greater, respectively).

**Conclusions:** The economic burden of aspergillosis is substantial. Patients with aspergillosis utilize significantly more healthcare resources and thus incur greater healthcare costs than do similar patients without aspergillosis.

## BACKGROUND

Aspergillosis refers to a number of diseases, including allergic bronchopulmonary aspergillosis, pulmonary aspergilloma and invasive aspergillosis, caused by infection by the commonly-encountered fungus *Aspergillus*.[1] Though harmless to most, the fungus is responsible for considerable amounts of morbidity and mortality among immunocompromised populations, including those with chronic lung conditions, such as pneumonia and cystic fibrosis, cancer and human immunodeficiency virus (HIV), as well as those who have undergone bone marrow or solid-organ transplants.[2] Case fatality is estimated to be as high as 60 to 100% in immunocompromised populations.[2-4]

Currently, voriconazole and amphotericin B deoxycholate (D-AMB) are the only therapies licensed in the United States for primary treatment of invasive aspergillosis.[5] Voriconazole is a triazole antifungal medication administered via oral tablets or injection. Due to the superior clinical efficacy[6] and safety advantages associated with its use as an initial treatment, voriconazole has emerged in recent years as the standard of care in the primary treatment of invasive aspergillosis. Other compounds (lipid formulations of AMB, itraconazole, caspofungin) are indicated as salvage therapies or for prophylaxis (posaconazole) in some transplant patients and in some cancer patients at high risk for invasive aspergillosis.[3, 5]

Data show that hospitalizations for aspergillosis have increased over the last 30 years and that patients hospitalized with aspergillosis have longer average lengths of stay and higher rates of mortality than patients hospitalized with other illnesses.[7] The hospital costs of patients with an aspergillosis infection vary widely depending on the severity of infection, especially in the context of comorbidities. Patients with a secondary diagnosis of aspergillosis in hospital records, presumably indicative of an immunocompromised patient, have been noted to have greater mortality, longer inpatient length of stay, longer duration of antifungal therapy and higher total hospital costs than those with a principal diagnosis of aspergillosis.[5] Hospital charges for treatment of aspergillosis reported in the literature range from a median of approximately $40 000 for HIV patients[5] to $413 000 for individuals with a bone marrow transplant.[8] The proportion of the total hospital costs attributable to intravenous antifungal drugs is estimated to be 7.2%.[9]

While aspergillosis is most often treated in the hospital setting, the availability of effective oral antifungals such as oral voriconazole may be shifting some care to the outpatient setting, whether as initial care or as continuation of therapy initiated in the hospital setting. As such, it is important to understand treatment received and costs incurred outside of the hospital. To date, most available data on the treatment and costs of aspergillosis largely focus on the hospital setting. To address the gap in the literature, the objectives of this study are twofold: 1) to describe the outpatient antifungal treatment and healthcare utilization of patients diagnosed with aspergillosis and 2) to provide recent estimates of total and outpatient healthcare costs of patients with aspergillosis in the United States.

## METHODS

### Study Design

This retrospective, observational study evaluates the incremental economic burden of aspergillosis within a commercially-insured population in the United States. Administrative claims data were used to identify a study population of enrollees (employees and their dependents) with aspergillosis and a propensity score-matched comparison group without aspergillosis. Healthcare utilization and expenditures were evaluated over a variable-length follow-up or post-index period. The economic burden of aspergillosis was estimated as the difference in the mean annualized costs between aspergillosis cases and controls, adjusted for differences in patient characteristics and variable patient follow-up.

### Data Sources

All patients who met the study eligibility requirements in the Truven Health Analytics' MarketScan® Commercial Claims and Encounters (Commercial) and Medicare Supplemental and Coordination of Benefits (Medicare) Research Databases were selected into the study. The Commercial database includes medical and pharmacy claims data from a variety of fee-for-service and managed care health plans, including exclusive provider organizations

(EPO), preferred provider organizations (PPO), point of service plans (POS), indemnity plans, health maintenance organizations (HMO), and most recently consumer driven health plans (CDHP). The Medicare database includes data from both Medicare-covered and employer-covered payments. These databases include enrollment data from approximately 150 large employers and health plans across the United States, which provide private healthcare coverage. More than 60 million employees, dependents, and retirees are included in these databases in the last three years. Both the MarketScan Commercial and Medicare Databases provide detailed cost, use, and outcomes data for healthcare services provided in both the inpatient and outpatient settings. The inpatient and outpatient medical claims are linked to outpatient pharmacy claims and person-level enrollment data through the use of unique enrollee identifiers.

**Study Sample and Inclusion Criteria**

Patients included in the aspergillosis study population were adults age 18 and older with at least one inpatient admission, emergency room (ER) or outpatient visit with a diagnosis of aspergillosis (*International Classification of Diseases, 9th Revision, Clinical Modification* diagnosis code 117.3 or 484.6) between July 1, 2004 and March 1, 2011. The patient's index date was the date of the first claim indicating a diagnosis of aspergillosis during the study period. All patients were required to have at least six months of continuous pre-index and at least one month of continuous post-index health plan and pharmacy benefit enrollment. Patients with a diagnosis of aspergillosis in the pre-index period were excluded. To maximize use of available data, the follow-up period was variable in length, and patients were followed until the end of their continuous enrollment or March 31, 2011, whichever occurred first.

A sample of control patients without aspergillosis who also met the inclusion criteria were matched to aspergillosis patients using propensity score matching. Potential controls were selected from a 1:1000 demographically similar sample (matched on index year, age group, sex, region, and year of follow-up) of health plan enrollees without aspergillosis during the study period. The 5573 aspergillosis cases and 4 932 497 potential control patients were included in the propensity score matching process (described in detail below). Following the matching process, 5499 aspergillosis cases and an equal number of controls were included in the study population.

**Variable Definitions and Propensity Score Matching**

Patient demographic and clinical characteristics, identified during the 6-month baseline period, included age (as a continuous measure and categorized as follows: 18-34, 35-44, 45-54, 55-64, 65-74, and 75+), sex, urbanicity, U.S. Census region (South, Northeast, North Central, West, or data unavailable/unknown), index year (2004 to 2011), and health plan type (indemnity plans, PPO or POS plan, HMO and other capitated plans, high deductible health plans, and other). Index year was assigned based on the year of the patient's first observed aspergillosis diagnosis meeting all other inclusion criteria during the study period for the aspergillosis cases; for non-aspergillosis cases, the index date was randomly assigned based on the distribution of index dates in the aspergillosis cohort. A Charlson comorbidity index (CCI)[10,11] score (Deyo version) was calculated for the baseline period. The CCI estimates the burden of comorbid illness from diagnoses associated with chronic diseases, with higher scores indicating a greater probability of major disability or death due to comorbid illness in the 12 months after it was evaluated. In addition, a series of flags were created to identify underlying immunocompromising conditions observed during the pre- and separately, the pre- or post-index periods as follows: cancer, bone marrow transplant, hematopoietic stem cell transplant (HSCT), human immunodeficiency virus (HIV), lung transplant, and neutropenia. Flags were also created to indicate the presence of graft-versus-host disease (GVHD), diabetes, and pulmonary diseases during the pre- or post-index periods. Pre-index utilization of medications in the following classes was also evaluated: systemic corticosteroids, immunosuppressive medications, warfarin, and broad spectrum antibiotics. Finally, total healthcare expenditures during the pre-index period were calculated.

Propensity score matching was used to ensure a similar distribution of specific confounding characteristics between aspergillosis patients and a sample of matched controls.[12,13] The matching variables were: age group, gender, Census region, index year, the presence of a pre-index diagnosis of cancer, bone marrow transplant, HSCT, lung transplant, neutropenia, or HIV, total pre-index healthcare costs, and number of days of follow-up. To perform the propensity score matching, logistic regression was used to predict the probability of having aspergillosis, using the values of various matching variables. Aspergillosis and non- aspergillosis patients were matched separately within the Commercial and

Medicare cohorts on the basis of the propensity score using a nearest neighbor metric to establish the final 1:1 match. Standardized differences (a function of mean values and standard deviations in the cases and controls) less than 10 for the majority of matching factors were considered an acceptable match.[14]

### Outcome Variables and Statistical Analyses

Outpatient utilization during the post-index period of antifungal therapy, which may be used to treat patients with aspergillosis (amphotericin B, an echinocandin (anidulafungin, caspofungin, micafungin), itraconazole, posaconazole, and voriconazole) was evaluated, as was duration (in days) of the first observed antifungal therapy. Concomitant medication use was also evaluated, including use of systemic corticosteroids, immunosuppressive agents, broad spectrum antibiotics and chemotherapeutic agents (see Table 3).

Healthcare utilization and expenditures were evaluated for the post-index period for aspergillosis cases and matched controls. Total healthcare expenditures in the post-index period included payments for inpatient admissions, outpatient visits and services (including ER visits, outpatient office visits, laboratory and radiology claims, and other outpatient services), and outpatient prescription drugs. Utilization and expenditures within each of these categories were also evaluated separately. All dollar metrics were inflated to 2011 values using the Medical Care Component of the Consumer Price Index (CPI). Healthcare utilization and expenditures were evaluated for the variable-length follow-up and results are reported both without adjustment for length of follow-up and in terms of per patient per month (PPPM).

Medical expenditures were first transformed using a common ($log_{10}$) transformation. Generalized linear models (GLM) were used to adjust for any remaining imbalances from matching to estimate the effects of aspergillosis diagnosis on $log_{10}$ transformed total and outpatient healthcare expenditures. As the length of patient follow-up in this study was variable, it was necessary to accommodate this feature of the study and reduce the potential bias that could be introduced by using a summary of costs, post index.[15]

A variation on a procedure suggested by Etzioni, *et al.*,[16] was employed to appropriately adjust the costs under the condition of incomplete or varying follow-up. Briefly, the probability of remaining in the study to the beginning of a time interval was calculated with a Kaplan-Meier/Product Limit estimator and then multiplied by average costs over the interval (back-transformed to the original or dollar scale using a method suggested by Dambolena, *et al.*[17] conditional on remaining in the study to the start of the interval. The key independent variable was an indicator for aspergillosis status. The control variables included: age, gender, insurance type, region, index year, pre-index CCI score, number of unique ICD-9-CM diagnosis codes in the pre-index period, underlying immunocompromising conditions in the pre-index period (cancer, bone marrow transplant, HSCT, lung transplant, neutropenia, HIV), total medical costs in pre-period and number of days of follow-up. Patients without aspergillosis served as the reference group; the effects of an aspergillosis diagnosis on transformed total and outpatient healthcare costs were assessed relative to controls without aspergillosis.

## RESULTS

The demographic and clinical characteristics of aspergillosis cases and the propensity score-matched controls (n=5499 for each cohort) are presented in Tables 1 and 2, respectively. As shown in the tables, the propensity score matching procedure resulted in comparable groups with only one of the matching variables yielding a standardized difference of >10 between the cases and controls (the standardized difference for age within the Medicare study population was 11). Post-matching, the average age of the aspergillosis study cohort was 57.8±15.3 years, and 48.6% were female. One-third (n=1860) of the overall case study population was identified from the Medicare Database and 95% (n=1762) of these patients were aged 65 and older. Two-thirds of the aspergillosis cases had their index diagnosis in the outpatient setting. The three most common immunocompromising comorbidities observed during the pre- or post-index periods for aspergillosis cases and controls, respectively, were cancer (64.2% and 60.6%), pulmonary diseases (63.4% and 27.8%) and diabetes (24.6% and 26.9%).

### Outpatient Treatment

Table 3 presents the outpatient prescription drug utilization of the study population during the evaluation period. Initial outpatient antifungal therapy observed within 30 days of the index diagnosis included: voriconazole (19.8%), itraconazole (7.5%), an echinocandin (i.e., caspofungin, micafungin, or anidulafungin) (2.4%), amphotericin B (1.5%) and posaconazole (0.90%). Few patients were treated with more than one antifungal within 30 days of index (0.42%), while no outpatient antifungal therapy within 30 days of the index diagnosis was observed for more than two-thirds of the study population (67.5%). One-quarter (25.2%) of the aspergillosis study population had at least one prescription claim for an antifungal after the 30 day period following index. The average duration of antifungal therapy during the post-index period was 51.8 ± 80.1 days. Initial outpatient treatment with voriconazole was significantly longer (mean = 60.6 ± 91.1 days; $p < 0.001$) than outpatient treatment with amphotericin B (19.6 ± 37.4) and the echinocandins (4.5 ± 12.9) and similar to the duration of treatment with posaconazole (47.1 ± 51.0) and itraconazole (53.6 ± 63.3) (data not shown, available from the authors upon request). Nearly three-fourths (73.0%) of the aspergillosis study population had at least one outpatient prescription claim for a systemic corticosteroid during the post-index period, 35.3% had at least one claim for a chemotherapeutic agent, and 11.2% had at least one claim for an immunosuppressive medication.

**Table 1.** Characteristics of Aspergillosis Cases and Propensity Score-Matched Controls

| | Cases | Controls | p-values |
|---|---|---|---|
| **Number of patients** | 5,499 | 5,499 | |
| **Age, in years (mean, SD)†** | 57.8 (±15.3) | 58.3 (±15.4) | 0.088 |
| **Female (%)†** | 48.60% | 48.40% | 0.79 |
| **Geographic Region (%)†** | | | 0.87 |
| Northeast | 9.00% | 8.90% | |
| North Central | 28.10% | 28.00% | |
| South | 36.60% | 37.40% | |
| West | 25.40% | 25.00% | |
| Unknown | 0.90% | 0.70% | |
| **Urban Residence (%)** | 85.50% | 82.80% | 0.0002 |
| **Health Plan Type (%)** | | | 0.92 |
| Indemnity plan | 20.50% | 20.30% | |
| HMO/other capitated plan | 17.80% | 17.50% | |
| PPO/POS | 57.10% | 57.50% | |
| High deductible plan | 2.60% | 2.80% | |
| Other/unknown | 1.90% | 1.90% | |
| **Index Year (%)†** | | | 0.74 |
| 2004 | 8.60% | 8.40% | |
| 2005 | 13.00% | 13.60% | |
| 2006 | 9.80% | 9.10% | |
| 2007 | 13.30% | 12.90% | |
| 2008 | 17.10% | 17.20% | |
| 2009 | 17.90% | 17.50% | |
| 2010 | 18.00% | 18.70% | |
| 2011 | 2.20% | 2.40% | |

HMO=health maintenance organization; PPO=preferred provider organization; POS=point of service plan; †
Variables included in the propensity score matching algorithm; all variables had standardized differences of less than 10 between the aspergillosis cases and controls with the exception of age for the Medicare population (standardized difference = 11).

**Table 2.** Pre-Index Clinical Characteristics of Aspergillosis Cases and Propensity Score-Matched Controls

| | Cases | Controls | p-values |
|---|---|---|---|
| **Pre-Index CCI score (mean, SD)** | 2.0 (±2.3) | 2.0 (±2.6) | 0.93 |
| **Unique ICD-9-CM diagnosis codes (mean, SD)** | 15.2 (±10.8) | 12.5 (±10.1) | <0.0001 |
| **Pre-index comorbidities (%)†** | | | |
| Cancer | 39.10% | 40.30% | 0.192 |
| Bone marrow transplant | 2.30% | 2.40% | 0.704 |
| HSCT | 0.90% | 0.90% | 0.839 |
| HIV | 0.90% | 1.00% | 0.922 |
| Lung transplant | 3.00% | 2.90% | 0.734 |
| Neutropenia | 13.30% | 14.90% | 0.016 |
| **Pre- or post-index comorbidities (%)** | | | |
| Cancer | 64.20% | 60.60% | <0.0001 |
| Bone marrow transplant | 5.20% | 3.10% | <0.0001 |
| HSCT | 2.00% | 1.30% | 0.0058 |
| HIV | 1.20% | 1.10% | 0.591 |
| Lung transplant | 8.50% | 4.80% | <0.0001 |
| Neutropenia | 20.40% | 17.80% | 0.0007 |
| GVHD | 1.90% | 0.90% | <0.0001 |
| Diabetes | 24.60% | 26.90% | 0.0056 |
| Pulmonary diseases | 63.40% | 27.80% | <0.0001 |
| **Pre-index medication use (%)** | | | |
| Systemic corticosteroids | 67.30% | 40.70% | <0.0001 |
| Immunosuppressive medications | 9.80% | 6.00% | <0.0001 |
| Warfarin | 6.80% | 9.70% | <0.0001 |
| Broad spectrum antibiotics | 65.40% | 43.30% | <0.0001 |
| **Total costs in pre-index period (mean, SD)†** | $67 752.7 (±$132 666) | $69 376.7 (±$218 034) | 0.637 |

SD=standard deviation; CCI=Charlson Comorbidity Index; HSCT=hematopoietic stem cell transplantation; HIV=human immunodeficiency virus; GVHD=graft-versus-host disease; †Variables included in the propensity score matching algorithm; all variables had standardized differences of less than 10 between the aspergillosis cases and controls

### Healthcare Utilization and Unadjusted Healthcare Expenditures

As shown in Table 4, a higher proportion of aspergillosis cases had an inpatient admission (49.5% vs. 37.1%), as well as a longer average length of stay (9.5±13.6 vs. 8.0±12.8 days), during follow-up than patients without aspergillosis. Aspergillosis cases also had a higher rate of ER visits (50.7% vs. 42.3%) and more per PPPM outpatient office visits (1.3±1.4 vs. 0.9±1.1), laboratory tests (4.0±11.0 vs. 2.4±5.8) and outpatient prescriptions (3.8±3.4 vs. 3.1±2.9) compared to patients without aspergillosis. All comparisons were significant at p<0.001.

As shown in Table 5, patients with aspergillosis had significantly higher PPPM expenditures in the post-index period compared to matched controls in every category of service. Mean unadjusted total PPPM healthcare expenditures were $8840±$21 853 for aspergillosis cases, compared to $5726±$26 569 for the controls, while the mean unadjusted PPPM outpatient prescription expenditures were $881±$1361 for the aspergillosis cases, compared to $517±$1663 for the controls. Outpatient costs (visits, services, and prescriptions) accounted for approximately 44% of the total costs of patients with aspergillosis.

**Table 3.** Post-Index Outpatient Antifungal and Other Therapies of Aspergillosis Cases

| | **%/Mean (SD)** |
|---|---|
| **Antifungal treatment in first 30 days of index (%)** | |
| Amphotericin B | 1.50% |
| Voriconazole | 19.80% |
| Itraconazole | 7.50% |
| Posaconazole | 0.90% |
| Echinocandins | |
| Caspofungin | 1.90% |
| Micafungin | 0.50% |
| Anidulafungin | 0.10% |
| Treated with more than 1 Antifungal at index | 0.40% |
| No observed treatment at index | 67.50% |
| **Duration of first observed antifungal therapy (mean, SD)** | 51.8 (80.1) |
| **Antifungal therapy >30 days of index (%)** | 25.20% |
| Amphotericin B | 2.20% |
| Voriconazole | 15.50% |
| Itraconazole | 7.90% |
| Posaconazole | 2.50% |
| Echinocandins | |
| Caspofungin | 1.90% |
| Micafungin | 0.80% |
| Anidulafungin | 0.10% |
| **Concomitant Medications, post-index period (%)** | |
| Amiodarone | 2.10% |
| Anti-retroviral therapy | 0.90% |
| Antituberculosis medications | 4.00% |
| Chemotherapeutic agents | 35.30% |
| Immunosuppressive medications | 11.20% |
| Phenobarbituates | 0.80% |
| Statins | 27.00% |
| Systemic corticosteroids | 73.00% |
| Warfarin | 9.50% |
| Therapeutic Drug Monitoring (%) | 24.20% |

SD=standard deviation

**Table 4.** Healthcare Utilization of Aspergillosis Cases and Propensity-Score Matched Controls

| | Cases | Controls | p-values |
|---|---|---|---|
| **Inpatient Admissions** | | | |
| Patients with an admission (%) | 49.50% | 37.10% | <0.0001 |
| PPPM number of admissions (mean, SD) | 0.1 (±0.2) | 0.1 (±0.2) | <0.0001 |
| Number of inpatient admissions (mean, SD) | 1.4 (±2.4) | 0.9 (±1.8) | <0.0001 |
| Length of stay (mean, SD) | 9.5 (±13.6) | 8.0 (±12.8) | <0.0001 |
| **Emergency Department Visits** | | | |
| Patients with an ER visit (%) | 50.70% | 42.30% | <0.0001 |
| PPPM number of ER visits (mean, SD) | 0.1 (±0.3) | 0.1 (±0.3) | <0.0001 |
| Number of ER visits (mean, SD) | 1.9 (±4.7) | 1.4 (±7.2) | <0.0001 |
| **Outpatient Physician Office Visits** | | | |
| Patients with a visit (%) | 91.60% | 90.50% | 0.0386 |
| PPPM number of visits (mean, SD) | 1.3 (±1.4) | 0.9 (±1.1) | <0.0001 |
| Number of visits (mean, SD) | 28.0 (±34.3) | 20.6 (±27.9) | <0.0001 |
| Ophthalmology visits | | | |
| Patients with a visit (%) | 14.40% | 11.70% | <0.0001 |
| PPPM number of visits (mean, SD) | 0.0 (±0.1) | 0.0 (±0.1) | 0.0541 |
| Number of visits (mean, SD) | 0.4 (±1.4) | 0.3 (±1.5) | 0.1271 |
| **Laboratory Tests** | | | |
| Patients with a laboratory test (%) | 87.50% | 83.20% | <0.0001 |
| PPPM number of laboratory tests (mean, SD) | 4.0 (±11.0) | 2.4 (±5.8) | <0.0001 |
| Number of laboratory tests (mean, SD) | 60.1 (±137.6) | 38.0 (±78.3) | <0.0001 |
| **Outpatient Prescriptions** | | | |
| Patients with an outpatient prescription (%) | 91.80% | 93.00% | 0.0130 |
| PPPM number of prescriptions (mean, SD) | 3.8 (±3.4) | 3.1 (±2.9) | <0.0001 |
| Number of prescriptions (mean, SD) | 88.2 (±116.3) | 72.8 (±102.5) | <0.0001 |

PPPM=Per Patient Per Month; SD=standard deviation

**Table 5.** Unadjusted Healthcare Expenditures of Aspergillosis Cases and Matched Controls

| | Cases | | Controls | | |
|---|---|---|---|---|---|
| | **Mean** | **SD** | **Mean** | **SD** | **p-value** |
| Inpatient admissions, total | $46 034 | $126 855 | $24 273 | $81 887 | <0.0001 |
| PPPM | $4664 | $17 583 | $2292 | $10 676 | <0.0001 |
| Emergency department | $1058 | $5989 | $759 | $3235 | 0.0010 |
| PPPM | $65 | $331 | $46 | $181 | <0.0001 |
| Outpatient physician office visits | $2891 | $3992 | $2076 | $3375 | <0.0001 |
| PPPM | $143 | $212 | $98 | $151 | <0.0001 |
| Ophthalmology visits | $38 | $138 | $32 | $144 | 0.0270 |
| PPPM | $2 | $8 | $1 | $6 | 0.0070 |
| Laboratory tests | $4444 | $14 978 | $2140 | $5766 | <0.0001 |
| PPPM | $345 | $1,717 | $150 | $505 | <0.0001 |
| Other outpatient services | $33 826 | $81 888 | $31 818 | $129 366 | 0.3310 |
| PPPM | $2494 | $7471 | $2637 | $23 528 | 0.7000 |
| Outpatient Prescriptions | $18 490 | $34 486 | $11 152 | $29 083 | <0.0001 |
| PPPM | $881 | $1361 | $517 | $1663 | <0.0001 |
| Antifungals | $5943 | $20 740 | $263 | $3256 | <0.0001 |
| PPPM | $451 | $1712 | $18 | $210 | <0.0001 |
| Total medical expenditures | $110 686 | $196 912 | $75 961 | $178 360 | <0.0001 |
| PPPM | $8840 | $21 853 | $5726 | $26 569 | <0.0001 |

Notes: Expenditures were accumulated in the variable-length follow-up period of at least 1 month. With the exception of the costs of other outpatient services and ophthalmology visits, all point estimates for aspergillosis cases were significantly higher than the controls at p<0.001; SD=standard deviation; PPPM=per patient per month

## Adjusted Healthcare Expenditures

Multivariate-adjusted mean and incremental total and outpatient healthcare expenditures are shown in Table 6. After adjusting for any remaining imbalances following propensity score matching, GLM models showed total adjusted annualized healthcare expenditures were $72 712.85 among aspergillosis cases compared to $46 032.63 for the controls (incremental costs: $26 680.21). Total adjusted annualized outpatient expenditures were $31 423.69 among aspergillosis cases compared to $22 175.81 for the controls (incremental costs: $9247.88).

**Table 6.** Multivariate-adjusted* Total and Outpatient Costs of Patients with Aspergillosis

| | **Mean** | **SE** | **95% Lower Confidence Limit** | **95% Upper Confidence Limit** |
|---|---|---|---|---|
| **Total Costs** | | | | |
| Aspergillosis cases | $72 712.85 | $1282.99 | $70 380.19 | $75 362.39 |
| Matched controls | $46 032.63 | $820.45 | $44 412.43 | $47 683.69 |
| Incremental costs | $26 680.21 | $1417.33 | $23 954.10 | $29 616.82 |
| **Total Outpatient Costs** | | | | |
| Aspergillosis cases | $31 423.69 | $657.44 | $23 169.61 | $32 810.05 |
| Matched controls | $22 175.81 | $515.45 | $21 177.76 | $23 169.61 |
| Incremental costs | $9247.88 | $766.25 | $7829.48 | $10 878.76 |

*Models adjusted for age group, gender, health plan type, region, index year, pre-index CCI score, number of unique three-digit ICD-9-CM codes, total medical costs in pre-index period, number of days of follow-up, and a series of comorbidities: cancer, bone marrow transplant, hematopoietic stem cell transplant, lung transplant, graft-versus-host disease, neutropenia, HIV, diabetes and pulmonary diseases; SE=standard error

The estimated median incremental total costs of patients with aspergillosis, adjusted for the probability of remaining in the study for a given duration of follow-up (via the Kaplan Meier/Product Limit estimator), were $22 732.99, $19 620.51 and $17 870.09 for durations of 90, 180, and 365 days of follow-up , respectively (see Figure 1). The estimated median incremental total outpatient costs of patients with aspergillosis, which were adjusted for the probability of remaining in the study for a given duration of follow-up time (via the Kaplan Meier/Product Limit estimator), were $7686.37, $6631.56 and $6120.40 for durations of 90, 180, and 365 days, respectively. Upon first consideration, the estimates of costs, adjusted for varying patient follow-up time via the method suggested by Etzioni, *et al.*, may seem counterintuitive insofar as the costs appear to decrease over time ($22 732.99, $19 620.51 and $17 870.09 for durations of 90, 180, and 365 days, respectively). These adjusted costs are estimated by the product of two factors. The first is the probability of remaining in the study to the next time interval. This quantity, by definition, will decline over time with loss to follow-up. The second is the costs averaged over a particular interval.

With aspergillosis, the largest changes in a patient's costs occur early (post-index), and then increase more slowly as a person will either succumb to consequences of the infection or are successfully treated. Thus, the adjusted costs, as a product of these two factors, represents a conflict between two quantities moving in opposing directions as time moves forward. If the rate at which patients exit the study grows more quickly than the rate of change in the costs as time moves forward, it becomes clear that the product, the adjusted costs, will decline (i.e., the probability, a number less than one, continues to decrease faster towards zero than the costs can accumulate away from their initial high value).

**Figure 1.** Total Healthcare Costs Adjusted for Covariates and Length of Follow-up Probability

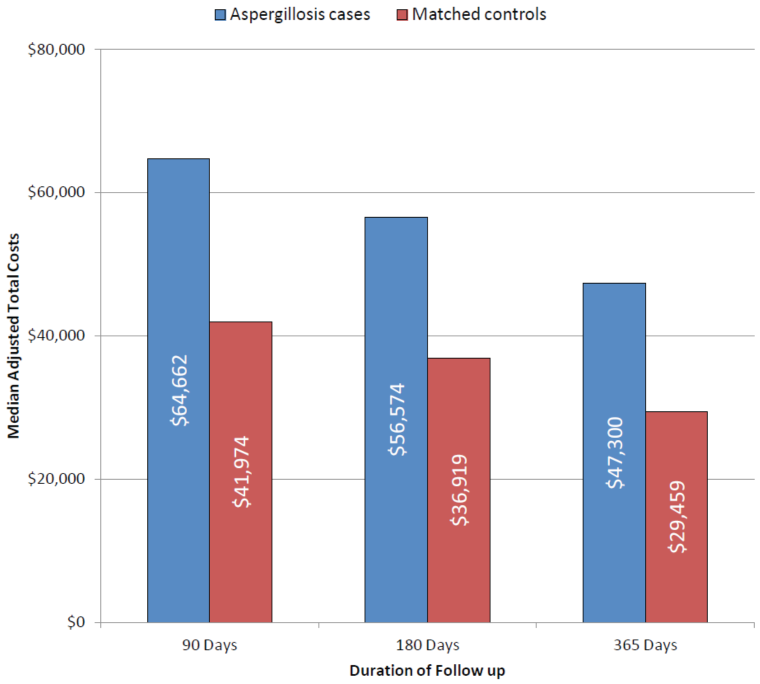

## DISCUSSION

This retrospective database analysis evaluated the total direct cost burden (i.e., inpatient and outpatient) and the outpatient antifungal treatment of patients with aspergillosis within a commercially-insured population in the United States. Healthcare expenditure, both total and outpatient expenditure, were substantially higher for patients with aspergillosis compared to those without. Using propensity score matched cohorts, GLM models showed that adjusted annualized incremental total and outpatient costs of patients with aspergillosis were $26 680.21 and $9247.88, respectively. As for outpatient antifungal therapy, we observed no outpatient prescription claim for an antifungal therapy within 30 days of the index diagnosis for two-thirds of the aspergillosis cases in our study; for those who did receive outpatient prescription therapy, voriconazole was the most commonly prescribed antifungal agent (60.9% of those treated).

This study provides recent estimates of the total cost burden of aspergillosis in the United States. Further, to our knowledge, it is the first to evaluate the total per patient outpatient burden, including physician office visits and laboratory and radiology services for patients with aspergillosis. Estimates of the outpatient costs of aspergillosis are important as treatment of the illness spreads to the outpatient setting with the availability of convenient, safe, and effective oral antifungal agents.

### Limitations

Certain limitations inherent to retrospective analyses using administrative claims data must be considered when interpreting these results. First, potential miscoding of medical claims and missing data are possible. Administrative data are collected for financial and administrative rather than research purposes, which can lead to suboptimal sensitivity and specificity when measuring conditions. Claims data arise from a person's use of a healthcare system and therefore depend on reimbursement coding practices from physician office visits, prescriptions filled and hospital stays. The second limitation is with respect to representativeness - while the MarketScan Research Databases represent a wide variety of nationally-representative employed patients and their dependents, it is not a random sample. The study population includes patients with Commercial and Medicare supplemental insurance, thus the results might not be generalizable to people with other insurance or with no insurance. An additional caveat to consider is that because our study design required at least 1 month of follow-up, the costs of patients who died within 1 month following diagnosis were not captured. As healthcare costs preceding death tend to be quite substantial, excluding such patients may have resulted in conservative incremental cost estimates in the present study. Finally, although we controlled for overt bias by propensity score matching aspergillosis cases and controls on a number of potentially key demographic and clinical characteristics, the two groups may differ on some unmeasured characteristics unrepresented by claims data.

## CONCLUSION

The total economic burden of aspergillosis continues to be substantial. Patients with aspergillosis utilize significantly more inpatient and outpatient healthcare resources and thus incur greater healthcare expenditures than do similar patients without aspergillosis. Efforts to improve prevention, diagnosis, and treatment of aspergillosis may help to reduce the cost of care and the overall patient burden associated with this illness.

## CONFLICT OF INTEREST DECLARATION

This research was sponsored by Astellas USA and was conducted by Truven Health Analytics. Publication of study results was not contingent on the sponsor's approval. Dr. Durden, Mr. Juneau, Ms. McMorrow and Mr. Fowler are employees of Truven Health Analytics; Dr. Chaudhari is an employee and stockholder of Astellas USA. Dr. Horn has received research funding from Astellas USA and has served as a consultant to and on advisory boards for Astellas USA.
